# Mapping the Salinity Gradient in a Microfluidic Device with Schlieren Imaging

**DOI:** 10.3390/s150511587

**Published:** 2015-05-20

**Authors:** Chen-li Sun, Shao-Tuan Chen, Po-Jen Hsiao

**Affiliations:** Department of Mechanical Engineering, National Taiwan University, 1 Roosevelt Road Section 4, Taipei 10617, Taiwan; E-Mails: hippo1347@gmail.com (S.-T.C.); r01522125@ntu.edu.tw (P.-J.H.)

**Keywords:** salinity gradient, microscale schlieren technique, microfluidic, quantitative analysis

## Abstract

This work presents the use of the schlieren imaging to quantify the salinity gradients in a microfluidic device. By partially blocking the back focal plane of the objective lens, the schlieren microscope produces an image with patterns that correspond to spatial derivative of refractive index in the specimen. Since salinity variation leads to change in refractive index, the fluid mixing of an aqueous salt solution of a known concentration and water in a T-microchannel is used to establish the relation between salinity gradients and grayscale readouts. This relation is then employed to map the salinity gradients in the target microfluidic device from the grayscale readouts of the corresponding micro-schlieren image. For saline solution with salinity close to that of the seawater, the grayscale readouts vary linearly with the salinity gradient, and the regression line is independent of the flow condition and the salinity of the injected solution. It is shown that the schlieren technique is well suited to quantify the salinity gradients in microfluidic devices, for it provides a spatially resolved, non-invasive, full-field measurement.

## 1. Introduction

Salinity has played an indispensable role in water quality, soil reclamation, ocean ecology, and even climate change [1,2,3,4]. Due to the increase of human consumption and irrigation, desalination becomes an important alternative to produce fresh water. With low energy consumption, membrane processes such as electrodialysis reversal (EDR) [5] or reverse osmosis (RO) [6] have grown popular among desalination technologies. Additionally, the reverse of these processes can be used in harvesting energy from the difference in salt concentration [7]. For pressure-retarded osmosis [8] or reversed electrodialysis [9], however, performance is limited by concentration polarization raised from the variation of concentration between the bulk solution and the membrane surface. In order to reduce concentration polarization, effective mixing near the membrane is desired [10]. On the other hand, in marine biology, salinity gradient is one of the major features in an estuary that has a profound effect on the diversity and ecology of microorganisms. Recent advance in microfluidics opens up the possibility to explore the influences of microscale salinity heterogeneity on microbial behavior at the level of individual cells [11]. Nevertheless, designing such microfluidic devices is accompanied with the growing need for accurate quantification of salinity inhomogeneity on a microscale. 

Conventional techniques to measure salinity of a solution include monitoring its specific gravity, electric conductivity, and refractive index. Boybay *et al.* [12] developed a microwave resonator system that was able to determine the composition of the test specimen by distinguishing the electrical properties of different materials. They showed that dissolved salt content affected the quality factor of the resonator so that salinity level between 0 and 100 g·kg^−1^ could be successfully discerned by sensing the reflection magnitude at the resonance frequency. Cong *et al.* [13] proposed an optical salinity sensor with a fiber Bragg grating (FBG) coated with hydrogels. When the hydrogel was immersed in the saline solution, the wavelength of the reflected “Bragg” signal shifted due to the mechanical stress induced within the hydrogel. Robinson *et al.* [14] designed a homogeneous, intensity modulated salinity sensor that consisted of a photonics crystal ring resonator (PCRR) and two inline quasi-waveguides. As seawater flowed through the device, the change in index of refraction led to the variation of the output power and a minimal discernable salinity of 1 g·kg^−1^ was attained. Also based on sensing the change in refractive index, surface plasmon resonance (SPR) was employed by Esteban, *et al.* [15], Díaz-Herrera *et al.* [16], and Kim *et al.* [17] to determine the degree of salinity. Esteban *et al*. [15] and Díaz-Herrera *et al.* [16] constructed a fiber-optic sensor and measured the attenuation of its transmitted power that had a linear relation with the refractive index of the medium. While their device was constrained to a point-wise detection, the approach of Kim *et al.* [17] was able to map the evolution of the salinity distributions in the near-wall region. With a SPR reflectance microscope, Kim *et al.* [17] established the correlation between the gray levels of the corresponding CCD pixels and the specified saline mass concentration. Despite their success of realizing label-free, full-field, and real-time measurements of salinity field with a spatial resolution of about 4 µm, the configuration of Kim *et al.* [17] required many optical components and a precise alignment for SPR angle adjustments and imaging. Another full-field detection of salinity was implemented by Kim *et al.* [18] in order to help develop a microfluidic device for continuous desalination. Kim *et al.* [18] used a fluorescence dye to track the process of ion concentration polarization but no quantitative information was given.

In this study, schlieren imaging is employed to facilitate non-invasive full-field measurements of salinity gradients in a microfluidic device. When salinity gradient is presented in the solution, the optical inhomogeneity leads to light deflection that can be detected by the schlieren imaging. The optical arrangement of the schlieren microscope is simple and easily established by placing a knife-edge in the back focal plane of the objective in order to produce local change in light intensity with respect to the gradient of refractive index. Although microscopic schlieren method is mainly used in qualitative investigations such as visualization of supersonic flow [19,20,21,22,23] or thermal jet [24], it is possible to obtain a relationship between the salinity gradients and the grayscale readouts of the micro-schlieren image. This follows a calibration procedure of mixing a saline solution with fresh water in a T-microchannel, and accurately mapping the salinity gradient in a target microfluidic device from the corresponding schlieren image. Herein, we choose the microscale schlieren method over the well-used differential interference contrast (DIC) microscopy because the intensity is not linearly proportional to the phase distribution for DIC. Despite many efforts to enable quantitative analysis for DIC [25,26,27,28,29], the available approaches usually require multiple DIC images taken from different shear direction and involve complicate post-processing calculation with iterative algorithms, which hinders measurements of unsteady phenomena. In contrast, microscale schlieren method is able to realize real-time full-field quantitation with a simple optical setup. This ability allows for an improvement in the design of a microfluidic device for studying microbial response to salinity change at cell level.

## 2. Experimental Setup

**Figure 1:**
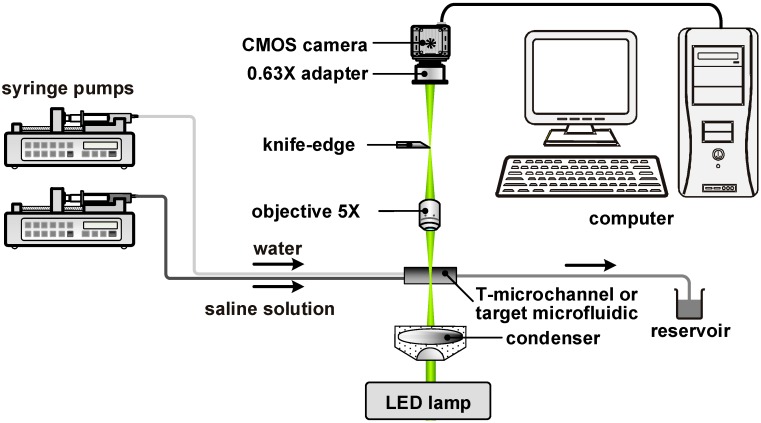
Experimental setup.

The schematic of the experimental setup for mapping salinity gradients in a microfluidic device is shown in Figure 1. The schlieren microscope is modified from a Hoffman modulation contrast microscope (DM IL LED, Leica Microsystems) [30] by removing the light slider and replacing the modulator with a simple knife-edge. For image acquisition, a CMOS camera (NX7-S1, Integrated Design Tools) is attached to the trinocular tube of the microscope, which is also connected to a personal computer. The cutoff of the knife-edge is set to 50% so that the measuring range is maximized with reasonable precision [31]. The knife-edge is oriented parallel to the main flow in the microfluidic device so that salinity gradient in the cross-stream direction is detected. The micro-schlieren images are recorded at 30 fps with a resolution of 1920 × 1080 pixels. With the 5× objective (N PLAN, Leica) and the 0.63× adapter, each pixel corresponds to a region of 2.3 × 2.3 µm^2^ in the microfluidic device. The LED lamp serves as the illumination source and is adjusted to produce the 8-bit image of the reference background with an average grayscale readout of 120. 

The microfluidic devices are made from transparent PDMS (Polydimethylsiloxane) material with a standard SU8 (SU-8 2151, MicroChem) molding method. A 1 mm thick glass-slide (S2215, Matsunami) is bonded to the PDMS layer after the oxygen plasma treatment. To eliminate the influences of light source and refraction caused by the microfluidic device itself, ratiometry is used so that each grayscale reading of a micro-schlieren image is divided by that of a reference image at the same pixel position. The reference image is taken when the microfluidic device is filled with pure water only and no optical inhomogeneity is present. To obtain the relationship between grayscale values and the salinity gradients, we use mixing of pure water and a saline solution in a T-microchannel as the benchmark. Both experiment and numerical simulation for mixing in the T-microchannel are performed, and the corresponding micro-schlieren images are then compared with the simulation results. The 170 µm deep T-microchannel comprises two 90 µm × 2500 µm feed channels and a 360 µm × 3000 µm exit channel. The saline solutions are prepared by mixing marine salt (hw-Marinemix professional, Wiegandt) with water. Besides sodium chloride, the marine salt also contains sulfur, calcium, and magnesium with compositions identical to natural seawater. The working fluids are dispensed into the microfluidic device by two syringe pumps (kds210, KD Scientific). At 25 °C, the salinity of seawater near the surface of the ocean is approximately 34.2 g·kg^−1^ [32]. For the calibration, we pick three different salinities, 10 g·kg^−1^, 20 g·kg^−1^, and 40 g·kg^−1^, for the saline solutions, and the Reynolds number at the outlet channel is set to 1 and 5 [33]. The concentration of the saline solution is checked by a density meter (DMA 4500 M, Anton Paar) and the relative uncertainty in salinity is ±0.2%. Although the composition of seawater varies, it is common practice in real applications to use the reference seawater as a standard in measurements of absolute salinity [34].

## 3. Working Principle

When a light ray passes through a medium with optical inhomogeneity, any gradient of refractive index perpendicular to the incident beam leads to light deflection. With a knife-edge positioned at the back focal plane of the objective, the bent light can either be partially intercepted to produce localized shadow or partially pass to produce localized brightness on a micro-schlieren image [35]. For a gradient of refractive index ∂*n*/∂*y*, the corresponding change in light intensity Δ*I* is proportional to the integral of the derivative of the refractive index over the medium thickness [36]:

(1) $$\frac{\Delta I}{I_{0}}\propto \int \frac{1}{n}\frac{\partial n}{\partial y}dz$$

where *I*_0_ is the reference light intensity and *z* is the coordinate parallel to the incident light. To accurately quantify ∂*n*/∂*y* from Δ*I*, however, we need to incorporate the influence of depth of field for microscale schlieren optics. Similar to the concept of depth of correlation for microscopic particle image velocimetry [37], optical homogeneity at various location along the optical axis contributes differently to the total intensity change on the resultant micro-schlieren image. Hence, the variation of light intensity on the micro-schlieren image can be assumed to be a weighted average of gradient of refractive index in different depth and Equation (1) is modified to:

(2) $$\frac{\Delta I}{I_{0}}\propto \int_{z-d/2}^{z+d/2}\frac{1}{n}\frac{\partial n}{\partial y}W\left(z\right)dz$$

where *d* is the medium thickness (*i.e.*, microchannel depth) and *W*(*z*) is the weighting function. Once *W*(*z*) and the relation between the refractive index and the salinity are known, we can use Equation (2) to determine the salinity gradient in the medium from the intensity variation on the micro-schlieren image.

## 4. Calibration Process

For seawater with a salinity *S* up to 43.5 g·kg^−1^, the refractive index varies linearly with salinity [38]. Therefore, ∂*n*/∂*y* in Equation (2) can be replaced by ∂*S*/∂*y* for *S* < 43.5 g·kg^−1^. For quantitative evaluation, it is necessary to correlate the salinity gradients with the grayscale readouts of micro-schlieren image. This is done by conducting a calibration process of mixing a saline solution with water in the T-microchannel (See *Experimental Setup*). Besides the micro-schlieren image of fluidic mixing, a reference image is also recorded when only water is fed to the T-microchannel and no optical inhomogeneity is present. On the other hand, the finite-element software package, COMSOL Multiphysics [39], can also be used to determine the three-dimensional salinity distribution in the T-microchannel—the flow is assumed to be steady, laminar, and incompressible. A constant diffusion coefficient is used that depends on the salinity of the incoming stream [40]. From the simulation results, the spatial derivative of salinity with respect to the cross-stream direction *y* for each *xy* plane is read and the average salinity gradient over the entire channel depth estimated. The variation of salinity gradient with *z* is negligible at with the tested Reynolds numbers (*Re* = 1 and 5). This is because diffusive mixing dominates mass transport for fluidic mixing in a T-microchannel under a Reynolds number of 140, despite the emergence of the Dean vortex [41]. Therefore, ∂*n*/∂*y* can be taken out of the integral in Equations (1) and (2), and a linear relation is expected between ∂*S*/∂*y* and *I*/*I*_0_:

(3) $$\frac{I}{I_{0}}=C_{1}+C_{2}\frac{\partial S}{\partial y}$$

where *C*_1_ and *C*_2_ are constants.

**Figure 2:**
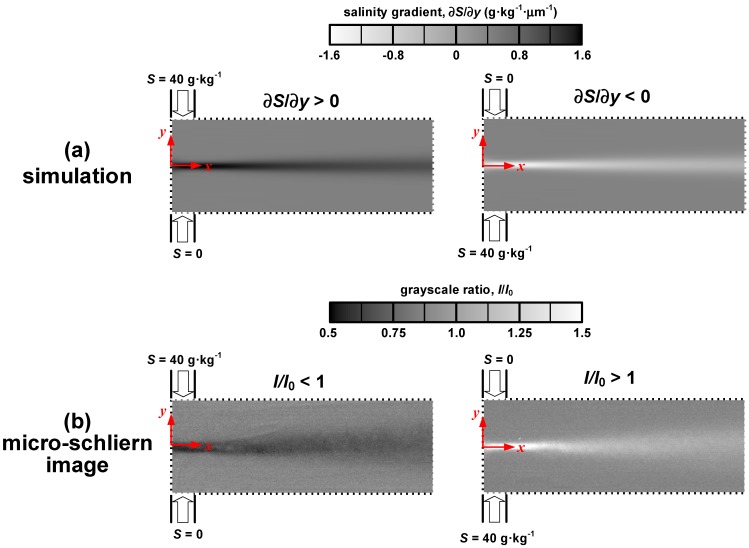
(**a**) Distributions of salinity gradient (numerical simulation); (**b**) Grayscale ratios (micro-schlieren images) in the T-microchannel, *Re* = 5.

The micro-schlieren images with the distributions of salinity gradient in the T-microchannel from simulation for *Re* = 5 and *S* = 40 g·kg^−1^ are compared (Figure 2). In the left panel (Figure 2), the salinity solution is injected into the T-microchannel from the upper inlet, while the opposite is shown in the right panel (Figure 2). In this study, the knife-edge is placed such as to obstruct the light bending toward the +*y* direction. Since the refractive index of the saline solution is higher than that of water, light deflects in the direction of increasing salinity. As a result, positive salinity gradient leads to a dark band, whereas negative salinity gradient produces a light band. The broadening band along the streamwise direction is ascribed to the dispersion effect. In the downstream region, salinity gradient diminishes and the schlieren band blurs. Although the distributions of salinity gradient and the micro-schlieren images exhibit great similarity, the simulation predicts a weaker dispersion effect than the experiments. Two factors may contribute to this deviation: interdiffusion between different species presented in our saline solution and the variation of diffusion coefficient with concentration. On one hand, ionic interactions lead to modification of the fluxes of diffusing ions and affect the relative distribution. When the mixing interface establishes in the T-microchannel, gradients of MgCl_2_ and Na_2_SO_4_ produce electric field that drives the fluxes of the ample Na^+^ and Cl^−^ ions, resulting in an enhancement of dispersion [42]. On the other hand, the diffusion coefficient of NaCl increases 4.1% as salinity reduces from 40 g·kg^−1^ to 0 [40], promoting diffusion near the edge of the band. Indeed, the simulation model can be improved by considering the multi-component diffusion. Nevertheless, it remains difficult to account for the contribution of different constituents to the change in local refractive index. For the purpose of detecting salinity gradient, we decide to treat seawater as a first-order composition system in this study [43].

**Figure 3:**
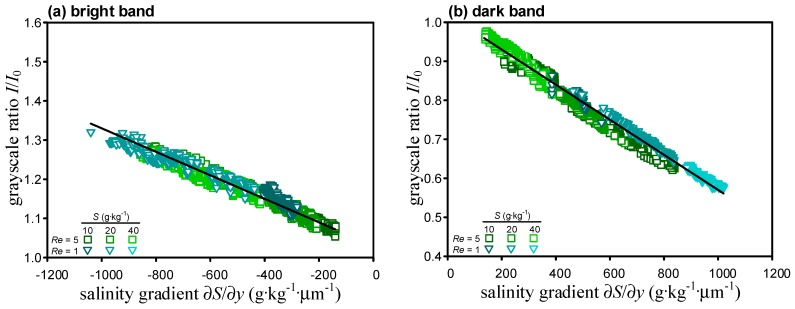
Variations of grayscale ratio with salinity gradient for (**a**) ∂*S*/∂*y* > 0 and (**b**) ∂*S*/∂*y* < 0.

After repeating the calibration process for different Reynolds number and salinity, the grayscale readouts from the micro-schlieren images and the values of the salinity gradient along the centerline of the T-microchannel (*y* = 0) are extracted and plotted (Figure 3). Here, ∂*S*/∂*y* < 0 and ∂*S*/∂*y* > 0 correspond to the bright and dark bands, respectively. It is seen that data points lie close to a straight line regardless of the Reynolds number and the salinity of the working fluid, suggesting the variation of grayscale ratio with salinity gradient is indeed independent of the flow condition as Equation (3) suggests. A regression analysis finds that *C*_1_ = 1.03, *C*_2_ = −3.0 × 10^−4^ kg µm g^−1^ for *I*/*I*_0_ > 1 and *C*_1_ = 1.02, C_2_ = −4.5 × 10^−4^ kg·µm·g^−1^ for *I*/*I*_0_ < 1 in Equation (3). The coefficients of determination both exceed 0.95, affirming that the linear model provides excellent fits. The slope of ∂*S*/∂*y* > 0 is steeper than that of ∂*S*/∂*y* < 0 (Figure 3) so that the microscale schlieren method has better sensitivity in the dark band region herein.

**Figure 4:**
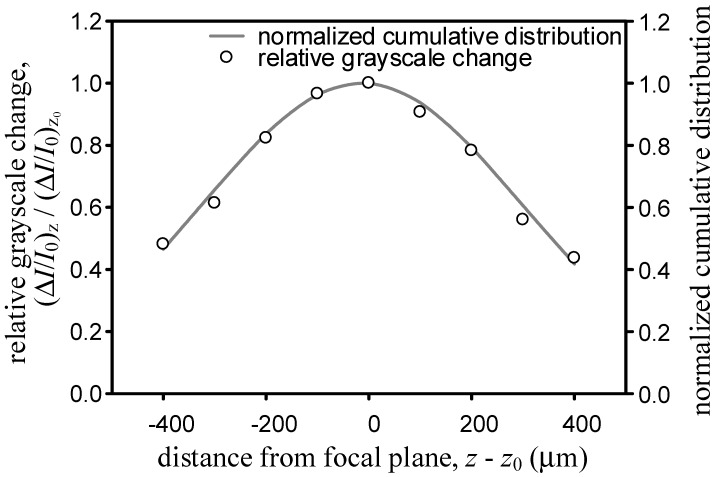
Comparison of relative grayscale change to the normalized cumulative distribution for the 5× objective.

Before applying Equation (3) with the determined *C*_1_ and *C*_2_ to map the salinity gradient in a target microfluidic device from the corresponding micro-schlieren image, the influence of channel depth needs to be corrected if the target microfluidic device has a different depth to that of the T-microchannel used in calibration. To account for the out-of-focus effect, the weighting function *W*(*z*) is approximated as a Gaussian distribution in Equation (2):

(4) $$W\left(z\right)=\exp\left[-{\left(z-z_{0}\right)}^{2}/2\sigma^{2}\right]$$

where *z*_0_ denotes the location of the focal plane and σ is the standard deviation of the Gaussian distribution. Therefore,

(5) $$\frac{\Delta I\left(z\right)}{I_{0}\left(z\right)}\propto \frac{\partial S}{\partial y}\int_{z-d/2}^{z+d/2}\exp\left[\frac{-{\left(z-z_{0}\right)}^{2}}{2\sigma^{2}}\right]dz$$

where *d* is the channel depth. To find the value of σ, the coaxial fine drive of the microscope is adjusted and the micro-schlieren images of fluidic mixing in the T-microchannel located at 11 different z-positions acquired. First, the normalized grayscale changes Δ*I*/*I*_0_ for each *z*, is calculated then the normalized grayscale change of the out-of-focus image is divided by those of the in-focus image and finally the values from 500 µm ≤ *x* ≤ 800 µm at *y* = 0 averaged (Figure 4). If the integral in Equation (5) is used to incorporate the cumulative contribution over the depth of the T-microchannel *d*_T_ located at a given *z*, the normalized cumulative distribution for a given σ can be estimated:

(6) $$\text{normalized cumulative distribution}=\int_{z-d_{\text{T}}/2}^{z+d_{\text{T}}/2}W\left(z\right)dz/\int_{z_{0}-d_{\text{T}}/2}^{z_{0}+d_{\text{T}}/2}W\left(z\right)dz$$

Through iteration, we find that σ = 308.5 µm leads to the smallest sum of squares of errors for the 5× objective. The line in Figure 4 depicts the regression outcome.

Once σ is known, Equation (3) is modified to include the out-of-focus effect. For σ = 308.5 µm, the integral in Equation (5) is equal to 167.8 µm for the T-microchannel. Hence, Equation (3) becomes

(7) $$\frac{I}{I_{0}}=C_{1}+\frac{C_{2}\int_{z-d/2}^{z+d/2}\exp\left[-{\left(z-z_{0}\right)}^{2}/2\sigma^{2}\right]dz}{167.8\;\text{μm}}\frac{\partial S}{\partial y}$$

where *d* is the depth of the target microfluidic device.

## 5. Quantitative Analysis

**Figure 5:**
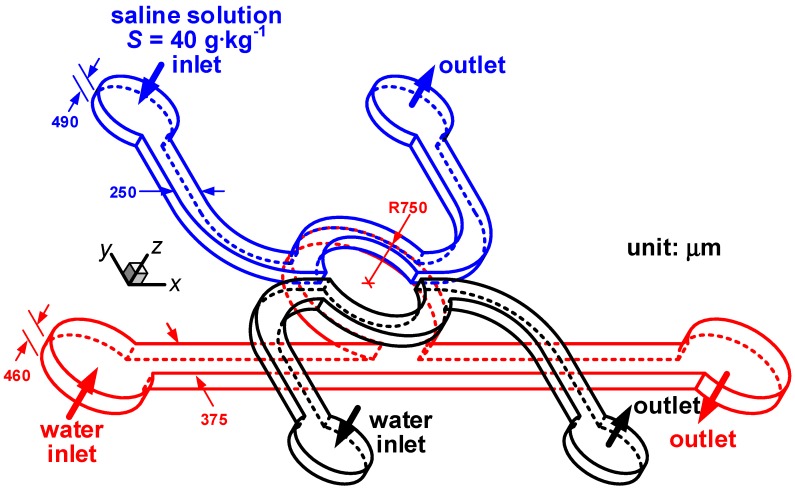
The microfluidic device for studying microbial response to a salinity gradient. The blue and black lines delineate the microchannels for saline solution and water in the top layer, respectively. The red lines outline the cavity-flow microchannel for water in the bottom layer.

Equation (7) provides a convenient conversion to map the salinity gradient distribution in a microfluidic device of a given depth *d* from the corresponding micro-schlieren image. To demonstrate its applicability, a microfluidic device that is designed for studying the response of marine microbes to spatial variation of salinity, is chosen as our target. As illustrated in Figure 5, the microfluidic device comprises two layers. The two microchannels in the top layer are 250 µm wide and 490 µm deep, where water and saline solution of 40 g·kg^−1^ are fed in order to produce salinity inhomogeneity in the cavity of the bottom-layer microchannel. The microchannel in the bottom layer is designed to introduce marine microbes, which comprises a 375 µm wide 460 µm deep straight microchannel connected to a cavity with a radius of 750 µm. The width of the cavity mouth is 551 µm. In this study, we focus on quantifying the salinity gradients in the microfluidic device so that no marine microbe is injected and only water is pumped through the microchannel in the bottom layer. The flow rate delivered to each inlet is modified so that the Reynolds numbers in all three microchannels are identical. In the definition of the Reynolds number, we use the hydraulic diameter of the rectangular duct as the characteristic length, *i.e.*, 331.1 µm for the two microchannels in the top layer and 413.2 µm for the microchannel in the bottom layer. Since mixing of saline solution and water occurs in the cavity, *d* equals to 460 µm in Equation (7). Therefore, the correlation between *I*/*I*_0_ and ∂*S*/∂*y* becomes

(8) $$\frac{I}{I_{0}}=C_{1}+2.5C_{2}\frac{\partial S}{\partial y}$$

Through Equation (8), the distribution of salinity gradient in the cavity is then quantified from the corresponding micro-schlieren image.

**Figure 6:**
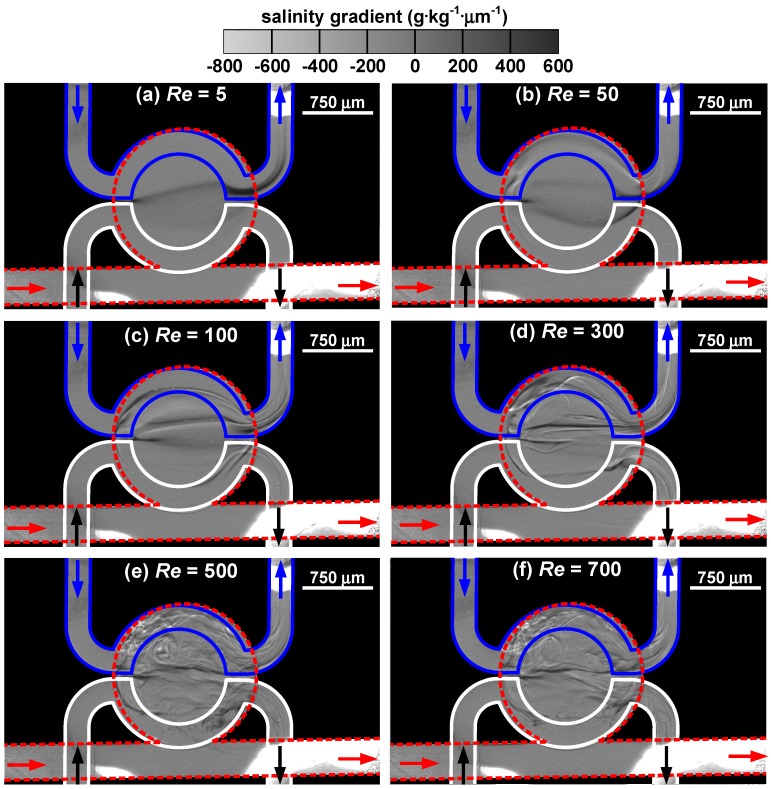
Distribution of salinity gradient in the target microfluidic device under different Reynolds numbers: (**a**) *Re* = 5, (**b**) *Re* = 50, (**c**) *Re* = 100, (**d**) *Re* = 300, (**e**) *Re* = 500, and (**f**) *Re* = 700.

The distributions of salinity gradient in the cavity of the target microfluidic device under various Reynolds numbers are shown in Figure 6. As the Reynolds number increases from 5 to 700, the flow rate varies from 0.097 to 13.576 mL·min^−1^ for water and from 0.102 to 14.273 mL·min^−1^ for saline solution in the top layer, and from 0.109 to 15.319 mL·min^−1^ for water in the bottom layer. At *Re* = 5, a faint arc spans across the cavity, suggesting that a positive salinity gradient (∂*S*/∂*y* > 0) is present in a direction perpendicular to the fluid flow in the straight channel. When the Reynolds number is low, fluid flow enters the cavity and follows its contour, inducing a counterclockwise-rotating vortex deep in the upper right corner of the cavity pocket. Hence, the dark ribbon bends slightly inward. Since the passages in the top layer lie over the edge of the cavity, the thicker rim region leads to a sudden darkening of the band. The trace of the band also suggests that water is somehow entrained into the exit channel of the saline solution. When the Reynolds number increases to 50, three distinctive threads emerge in the cavity. The bottom ribbon bulges toward the cavity mouth, whereas the top ribbon presses on the edge of the cavity pocket. It is found that white filaments appear near the two ends of the top ribbon, suggesting the presence of a three-dimensional flow field near the region that the saline solution enters or leaves the cavity. This helps to mix water with the saline solution and leads to the increase of folding and stretching fluids. At *Re* = 100, the three ribbons retain but become closer to each other. In addition, each ribbon consists of more than one strand and entrainment is found in both exit channels in the top layer. Due to the folding of fluid filaments, salinity field becomes more varying. The increase in fluid velocity reduces the diffusion transport and mixing occurs in a more advective manner. For *Re* ≥ 300, the flow field starts to exhibit irregularity. At *Re* = 300, the image reveals the streams coming from the top-layer channels strongly interfere with the salinity field in the cavity and distort the schlieren pattern. As *Re* increases to 500, small eddies significantly enhance mixing in the cavity and the presence of turbulence is apparent. At *Re* = 700, the schlieren pattern becomes less distinctive due to excellent homogenization.

This paper reports on the successful ability of microscale schlieren technique to map detailed distribution of salinity gradient in a microfluidic device. Although the proposed approach does not directly provide discrimination on constituents, it is possible to predict the concentration profiles of different species and account for composition anomalies of natural seawater by determine the interdiffusion coefficients through least squares fitting the refractive index profile across Taylor dispersion peak [42]. Despite its versatility, it is noted that microscale schlieren technique detects variation of refractive index, which can be affected by other independent variables such as temperature. The microscale schlieren technique expands the use of microfluidics in studies that are closely linked to microscale salinity patchiness, such as micro-organism interactions with a changing marine microenvironment or efficiency improvement of membrane processes for harvesting the blue energy.

## 6. Conclusions

In this study, the applicability of the microscale schlieren technique on detecting salinity gradient in microfluidics is explored. The major findings are summarized as follows.

1. A calibration procedure that conducts mixing water and a saline solution in a T-microchannel is employed to obtain the relation between salinity gradients and grayscale ratios. As long as the salinity of the solution is smaller than 40 g·kg^−1^, grayscale ratios of a micro-schlieren image vary linearly with salinity gradients. Moreover, we verify that this calibration line is independent of the Reynolds number in the T-microchannel and the salinity of the solution.
2. A weighting function, approximating as a Gaussian distribution, is introduced to incorporate the out-of-focus effect for microscale schlieren method when the target microfluidic device has a different depth than that of the T-microchannel. After correcting for the influence of the channel depth, the quantitation of salinity gradients in a microfluidic device that is designed to study the response of marine microbes to a change in microenvironment is demonstrated. The spatial variations of salinity gradient in turbulent mixing are captured by the micro-schlieren images at various Reynolds numbers.
3. The outcome of this study proves that spatially resolved, noninvasive, full-field measurements of salinity gradient in microscale can be realized using a schlieren microscope. The advances in quantitative analysis of salinity gradient will be beneficial to the studies of microscale transport phenomena near the porous membrane for blue energy harvesting and biological processes of the ocean in the future.
